# Revisiting the inverse Abel integral for reconstructing velocity-map images

**DOI:** 10.1039/d5cp00857c

**Published:** 2025-08-20

**Authors:** Chris Sparling, Jolijn Onvlee

**Affiliations:** a Institute for Molecules and Materials, Radboud Universiteit, Heyendaalseweg 135 6525 AJ Nijmegen The Netherlands c.sparling@hw.ac.uk j.onvlee@science.ru.nl

## Abstract

The velocity-map imaging (VMI) technique is used near ubiquitously throughout the study of gas-phase photophysics and chemical dynamics. Many VMI experiments rely on numerical reconstruction techniques to recover the full three-dimensional (3D) velocity distribution of photoproducts from the two-dimensional (2D) geometric projection – the Abel transform of the distribution – that is recorded in a typical experiment. The simplest mathematical approach for this reconstruction procedure is through use of the inverse Abel integral transform. Historically, though, this approach has performed poorly on real experimental data, and so the VMI community has devoted much effort into the development of alternative inversion strategies that avoid direct use of the integral. In this article, we challenge this firmly held belief, and show instead what advantages can be realised through this approach. Unlike many other competing approaches, the reconstruction technique presented here, which we refer to as the modified Abel integral transform (MAIT), does not require the lengthy pre-computation time for a large basis set or any manually adjustable regularisation parameters. Examples involving simulated and real experimental data are used to demonstrate the efficacy of our new approach. This method is shown to perform similarly to the most popular alternative strategies for extracting photoproduct angular distributions, and have a significant advantage over them when handling data with high levels of background noise, in particular.

## Abstract 2

A method for analysing velocity-map imaging data is presented. This approach, dubbed the Modified Abel Integral Transform, is easy to implement and particularly useful for handling data with high background noise levels.

## Graphical abstract

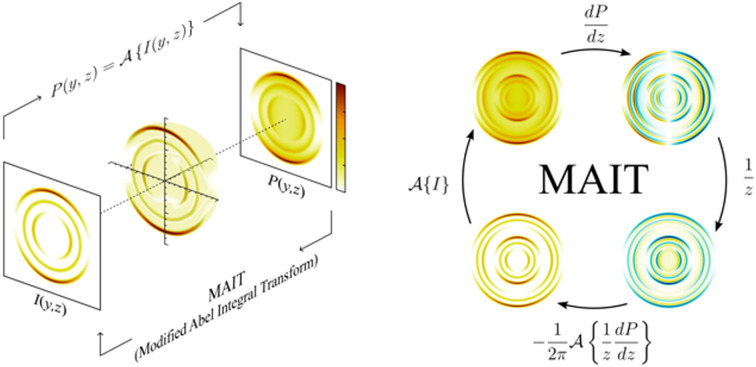

## I. Introduction

**Fig. 1:**
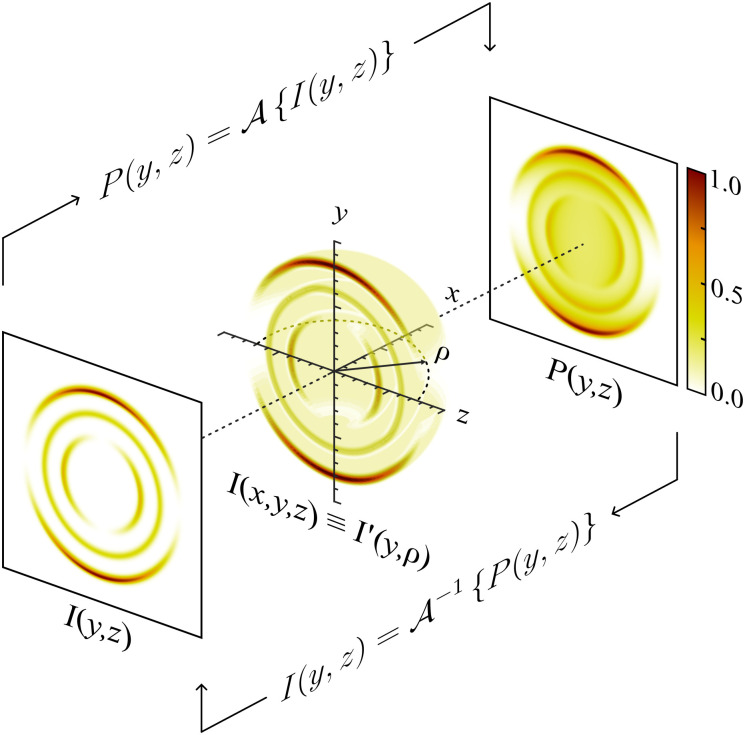
A schematic illustration of the forward and inverse Abel transform operations. A rendering of one hemisphere of a typical 3D-VMI distribution is shown in the centre, with its projection *P*(*y*,*z*) and central slice *I*(*y*,*z*) shown on the right and left, respectively. The forward Abel integral 
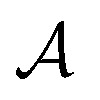
 transforms *I*(*y*,*z*) to *P*(*y*,*z*), and the inverse integral 
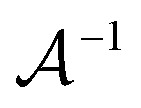
 transforms *P*(*y*,*z*) back to *I*(*y*,*z*).

Since their introduction in 1987,^1^ photoion/photoelectron imaging measurements have been used extensively for studying dynamics and photochemistry in the gas-phase.^2–6^ In subsequent years, numerous refinements and extensions to the original pioneering work of Chandler and Houston^1^ have established imaging firmly within the chemical physicists’ toolbox.^7–16^ Perhaps most notably, the introduction of velocity-map imaging (VMI) by Eppink and Parker in 1997^16^ allows for the high-resolution measurement of the complete (*i.e.*, all 4π-steradians) three-dimensional (3D) velocity distribution of photoproducts (ions or electrons) following some optical interaction. Although more advanced VMI spectrometers have been developed over the years to measure the entire 3D distribution (or a subsection of it) directly,^7–10,12,17–19^ in their most popular and simplest realisation, VMI spectrometers do not directly resolve the entire 3D distribution, *I*(*x*,*y*,*z*). Rather, they measure a two-dimensional (2D) projection of the distribution, *P*(*y*,*z*); effectively an integration of the distribution along the entire time-of-flight axis of the spectrometer (here, the *x*-axis – see Fig. 1):1
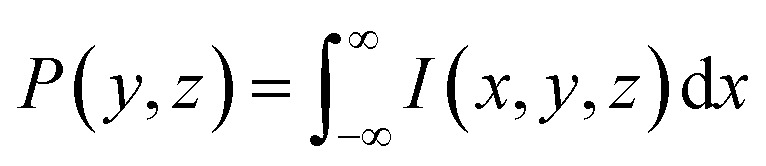


For unimolecular processes involving randomly oriented atoms/molecules interrogated solely with linearly or circularly polarised laser sources, the resulting 3D distributions will possess cylindrical symmetry about some axis (the polarisation direction for linear polarisation, or the optical propagation direction for the case of circular polarisations). For this special case, eqn (1) may be reframed in cylindrical coordinates as the Abel transform^2^ – the foundational equation for the analysis of VMI projection data.

It is defined by the integral:2
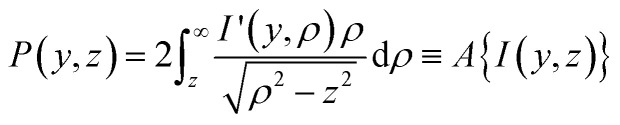
where *I*′(*y*,*ρ*) is a cylindrically symmetric distribution, and the radial distance *ρ* from the symmetry axis (here, the *y*-axis) in the *xz*-plane is 
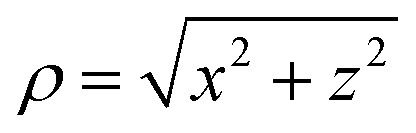
. Throughout the remaining equations in this article, 
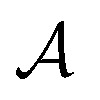
 will be used to denote the forward Abel transform. To instead reconstruct *I*′(*y*,*ρ*) [which is equivalent to *I*(*y*,*z*); the *yz*-planar slice through the full 3D distribution] from the measured image data *P*(*y*,*z*), the inverse Abel transform, 
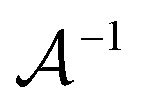
, is given by:^20^3
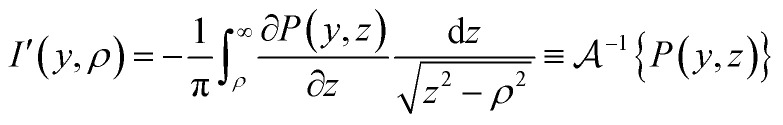


These different distributions and their relations to each other are illustrated in Fig. 1 – along with the definition of the coordinate system that will be used throughout this article. In principle, once *P*(*y*,*z*) is measured experimentally, eqn (3) allows for the full cylindrically symmetric 3D photoproduct distribution to be reconstructed perfectly. Only with this recovered distribution can the full volume of information about the system being studied with VMI be extracted from the data. As an aside, it is also possible to reconstruct 2D projections of non-cylindrically symmetric distributions, although these methods are far more complex and require either multiple distinct projection images,^21–25^ extensive numerical simulations^26–29^ or utilise novel machine-learning approaches.^30^ For many experimental applications, however, cylindrical symmetry is preserved in imaging data, allowing for the inverse Abel transform to be applied.

Some significant issues, though, begin to present themselves when one attempts to evaluate Abel's inverse integral directly with real (and imperfect) experimental data.^2^ A naïve numerical integration of eqn (3) leads to a large amount of noise (often several orders of magnitude larger than the physically meaningful reconstructed data) being directed toward the central symmetry axis of the distribution (the *y*-axis in Fig. 1), even for high-quality experimental data. The reason for this is two-fold: (i) the derivative in eqn (3) can amplify any statistical noise present across the experimental image data; and (ii) the 
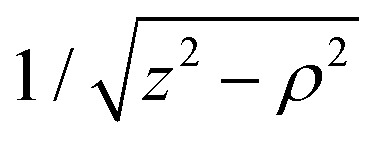
 factor in the integral further magnifies the integrand close to the centre line. The presence of this centre line reconstruction artefact makes the analysis of photoproduct angular distributions particularly challenging. Furthermore, solving the derivative required for the inverse Abel integral necessitates replacing the real data matrix with some numerical approximation which can be differentiated. This leads to unwanted ‘smoothing’ of the data and potentially impacts the final resolution of the reconstructed image. Due to these inherent problems, the simple inverse Abel integral has essentially been abandoned by the VMI community. Instead, much research effort has been devoted toward developing alternative approaches which are able to avoid any of the pitfalls of the basic inverse Abel integral. In 2019, many of the most popular strategies were compiled in PyAbel – a Python library for Abel transform functions.^31^ This excellent package allows for users to experiment with many different inversion approaches within a user-friendly and consistent environment. Interested readers are directed to this article^31^ for a review and detailed comparison of various inversion strategies, as only a brief overview will be given here.

Amongst the most popular forms of reconstruction algorithms are recursive methods, which iteratively find a solution *I*(*y*,*z*) which [when projected – using eqn (2)] matches best with the experimentally measured *P*(*y*,*z*).^32–36^ These can either use a simple linear least-squares approach in the optimization step^32,33^ or use more advanced maximum-likelihood models that can incorporate the correct Poissonian sampling statistics of noisy image data.^34–36^ Another family of inversion techniques is based on the so-called ‘onion-peeling’ algorithm.^37–41^ Starting at the edge of the image, contributions to *P*(*y*,*z*) coming from the *xz*-plane can be calculated and subtracted away. Repeating this at decrementing radii effectively ‘peels away’ the projected components of *P*(*y*,*z*), leaving behind only the central slice *I*(*y*,*z*).

In an alternative linear algebra-based approach, the BAsis Set EXpansion (BASEX) method introduced by Dribinski *et al.*^42^ expands the measured *P*(*y*,*z*) line-by-line as a sum of basis functions that have a known Abel inverse. By simply expanding the inverse functions using the basis coefficients calculated from the projected data, one arrives at the linear least-squares solution for *I*(*y*,*z*) without having to apply the inverse Abel transform directly to the imaging data. This gives rise to high-quality reconstructions, often with less centre line noise than the inverse Abel integral. Noise levels may be reduced even further by using matrix regularisation techniques [such as Tikhonov regularisation or truncated singular-value decomposition (SVD)] to stabilise the system of linear equations used in the inversion procedure, though the value used for these regularisation parameters are often chosen arbitrarily to make the most visually appealing reconstructions. The rapid matrix inversion approach,^43^ introduced to the VMI community by Livingstone *et al.*^44^ operates and performs similarly to BASEX and is extremely fast. This makes it perfect for processing large volumes of imaging data, such as is required in time-resolved photoelectron imaging experiments, for example.^45,46^

Both of these linear-algebra methods operate on the image data line-by-line; effectively treating each value of *y* independently. In an alternative approach, Garcia *et al.* proposed to instead fit to the whole image simultaneously using 2D basis functions in their polar coordinate formulation of BASEX, named pBASEX.^47^ This approach is possible because the angular structure of most typical VMI images is easily described by a small expansion of Legendre polynomials 
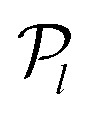
 in cos *θ*:^2,48^4
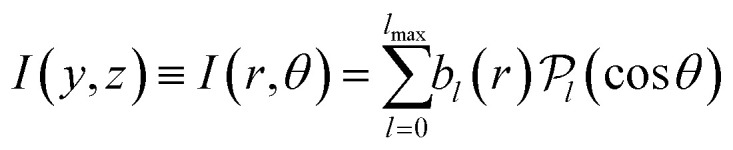
where *θ* is the emission angle of the photoproducts with respect to the symmetry axis of the distribution and 
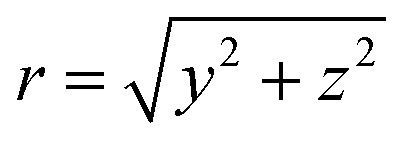
 is the radial coordinate in the *yz*-plane. The coefficients *b*_*l*_ are often referred to as anisotropy parameters and determine how much each Legendre polynomial term contributes to the full angular distribution. The upper bound on the expansion *l*_max_ is usually determined by the specific nature of the measured photofragmentation/ionization process. Constraining the reconstructed solution in this way reduces the presence of any reconstruction noise down to a single point at the image centre. Some onion-peeling algorithms also make uses of angular basis sets to fit to the reconstruction distribution at each peeling step.^39,40^ Similar results are also possible by using one-dimensional (1D) projections of these 2D basis functions in a formulation known as linBASEX.^49^

At time of writing, BASEX appears to be the most popular Abel inversion routine in use, with pBASEX being a close second (judging by citation records available from Web of Science). This is likely because versions of these algorithms have been in circulation in the community since the early 2000s and the use of simple matrix algebra operations makes them fast, direct (*i.e.* non-iterative) and intuitive. Basis expansion approaches do, however, come with the minor drawback that a basis set must be computed prior to the inversion of any experimental data. For the 1D functions used in BASEX, this procedure is not too time-consuming, but for the 2D functions of pBASEX the write times can be significantly longer, particularly for high-resolution images with complicated angular structures (although recent work has demonstrated that accelerated pBASEX basis write times are possible^50^). Alternatives to pBASEX that offer the same centre-line noise suppression qualities have been developed, but these either still require some form of pre-written basis function or have an extended reconstruction time per image.^39,40,51^ As such, research continues in the development and refinement of fast, basis set-free reconstruction routines for VMI data analysis.

This article presents one such method, that begins – perhaps surprisingly – with the unfashionable inverse Abel integral introduced in eqn (3). By simply multiplying and dividing by *z* inside the integral of eqn (3):5
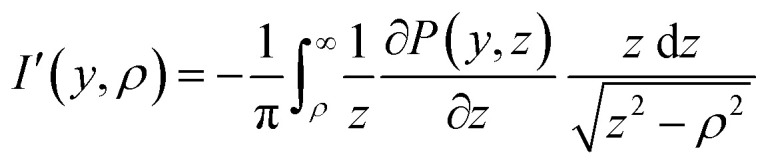
and rearranging some terms, the inverse transform may now be reframed as the *forward* (and far more numerically stable) Abel transform of another related function [after a change of variables and accounting for the factor of 2 in eqn (2)]:6
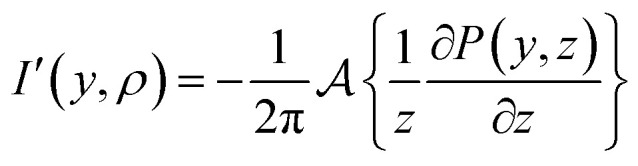


This publication will illustrate how, in part because of the properties this new intermediate function must possess and how fast and simple it is to calculate the forward Abel transform, this method – which we dub the modified Abel integral transform or MAIT approach – is far more suitable for VMI image reconstruction purposes than a naïve numerical integration of eqn (3). The following sections will discuss this in detail and illustrate with simulated and real data examples the performance of MAIT. It is shown how this method can achieve reconstructions of comparable quality to the basis set reconstruction approaches BASEX and pBASEX but without any regularisation parameters or the associated basis write time, which in the case of pBASEX can be several hours. This method also has some considerable advantages over these other approaches when handling imaging data with large levels of background noise.

## II. Numerical details

The derivative present in the inverse Abel integral [eqn (3)] is often cited as one of the primary motivations behind avoiding this approach altogether and developing alternative image reconstruction methods. This is because for real, pixelated and imperfect data, this derivative step can magnify any noise present in the image. When combined with the 
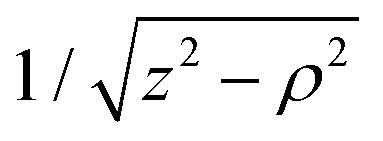
 factor required for the integration, this noise is amplified considerably toward the centre (*y*-axis) of the derivative image. By considering first a noise free distribution, we can see what properties the intermediate functions in eqn (6) posses.

**Fig. 2:**
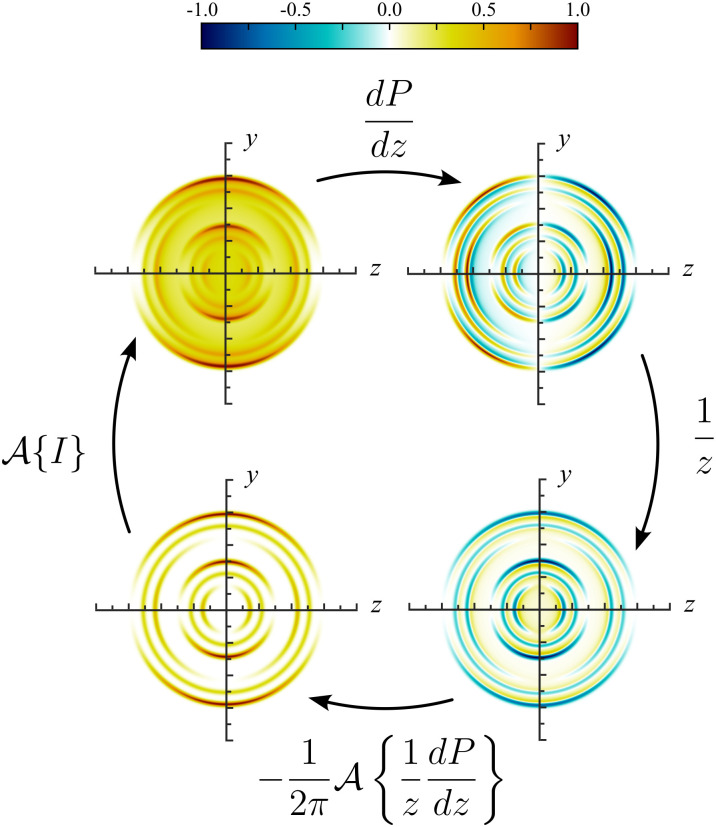
The steps of the MAIT image reconstruction strategy. Starting from the bottom left image and following the arrows clockwise. The projection of *I*(*y*,*z*) is described using the forward Abel transform, yielding *P*(*y*,*z*). After defining a new image by differentiating *P*(*y*,*z*) with respect to *z* and multiplying the result by 1/*z*, the original projection operation can be reversed by finding the Abel transform of the new image distribution.

Fig. 2 shows an example slice through a VMI distribution (bottom left) and the corresponding projection image that would be recorded in an experiment (top left). Upon differentiating with respect to *z* across the image, each projected ring in the simulated test image splits into an inner and outer ring [note the alternating red/blue (positive/negative) radial structure]. This numerical derivative is calculated simply using the built-in gradient function in MATLAB. While we find this produces acceptable results for each of our tested scenarios on real and simulated experimental data (see Section III A, D and E), it may have an impact on the reconstruction resolution of particularly narrow image features that are only a few pixels wide – as was mentioned briefly in the Introduction. This is discussed further in Section III C. Note that now, the derivative distribution is no longer symmetric about the *y*-axis like the original projection image, but is instead anti-symmetric (the left half of the image is the negative of the right half). This means that the angular structure of the derivative image may be described using an expansion of Legendre polynomials, analogous to eqn (4), but now with only odd-degree terms contributing.

These odd-degree polynomials must, by construction, be equal to zero along the symmetry axis (*y*-axis) of the distribution image. Therefore, the 1/*z* factor in eqn (5) will not necessarily introduce any infinitely large noise values to the intermediate function being Abel transformed. Rather, it results in a 0/0 indeterminate form. The true value the function takes as *z* approaches zero may be found algebraically. Since the derivative image is already an odd 2D function (given its anti-symmetry properties, see Appendix), it must already contain a factor of *z*. With this *z* factor divided out, the resulting image is shown in the lower right image in the cycle in Fig. 2. The inverse Abel transform of the image data *P*(*y*,*z*) can then be found by calculating the (far more numerically stable) forward Abel transform of the intermediate function and multiplying by 
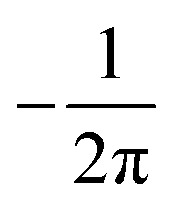
.

Since the forward transform is perfectly numerical stable, this step can be performed in principle using any established method. Here in this work, for speed and simplicity, we opt for the rapid matrix Abel transform developed for VMI analysis by Livingstone *et al*.^44^ This approach, discussed briefly earlier, models the Abel transform as a simple matrix multiplication. The matrix can be pre-computed ahead of time (like the basis sets matrices used in BASEX and pBASEX), but in practice this is often not necessary since it can be calculated very quickly on-the-fly.

As was mentioned briefly in the introduction [see eqn (4)], for multiphoton ionization and dissociation measurements there is a clear interpretation decomposing the projection image into Legendre polynomials. For example, in the laboratory measurement frame, the maximum number of Legendre polynomials *l*_max_ required to describe a photoproduct angular distribution *I*(*y*,*z*) resulting from an overall *N*-photon ionization/dissociation of randomly oriented atoms/molecules is 2*N*. Furthermore, only *even* degree polynomials typically contribute to the angular distribution, except for a handful of notable cases, such as the ionization of chiral molecules with circularly polarised light.^52–54^ When the derivative of the projection data is taken in the first step of the MAIT cycle, however, one additional Legendre polynomial term (resulting in 2*N* + 1 terms overall) must be included to properly describe the derivative image. Applying the 1/*z* factor in the following step of MAIT effectively divides this extra odd polynomial term back out again, and so the final Legendre polynomial content of the reconstructed *I*(*y*,*z*) image has *l*_max_ = 2*N*, as expected. The presence of only odd symmetry polynomial terms up to and including 2*N* + 1 in the derivative distribution can be used explicitly as a noise filter during the image reconstruction procedure, like the use of Legendre polynomials within pBASEX. We refer to this particular reconstruction procedure as the filtered modified Abel integral transform or fMAIT.

This polynomial expansion of the projection image, however, is more challenging for other experimental imaging techniques, such as molecular scattering,^27,55–57^ strong-field ionisation,^58–60^ and Coulomb explosion imaging,^13,61^ where highly anisotropic and/or structured images with very large or unknown values of *l*_max_ are commonplace. In these instances, it is still possible, of course, to proceed with this inversion procedure without the polynomial expansion step. The anti-symmetry of the derivative image can still be enforced by subtracting a copy of the derivative image mirrored about the *y*-axis from itself. This is implemented as standard in the MAIT and fMAIT algorithms. With this approach, a small amount of noise is present around the centre line of the final reconstruction, but this (as will be demonstrated thoroughly in Section III) amounts to a small level similar to the reconstruction noise typically present in images produced using BASEX.

**Fig. 3:**
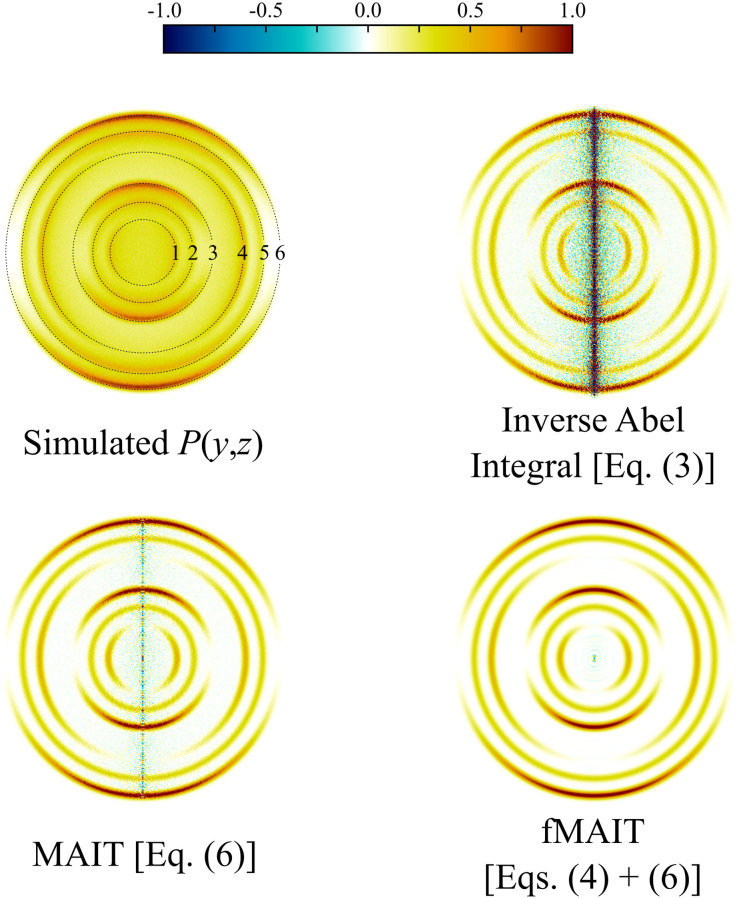
The varying reconstruction results possible by using different numerical approaches to find solutions to the inverse Abel integral for a simulated image. Even for relatively noise-free data, eqn (3) performs poorly. Eqn (6), on the other hand, presents with far less reconstruction noise. The numbered ring positions indicated in the top left panel will be used throughout the discussion in this article.

Fig. 3 shows an example simulated projection image (details on how this was generated are include in the following section) along with three reconstructions, all produced using different image reconstruction strategies. The top right reconstruction is produced by simply numerically integrating the inverse Abel integral [*i.e.*, eqn (3)] and results in a large amount of noise being directed toward the centre of the reconstruction, even for a high-quality projection image. The bottom left reconstruction is produced instead using eqn (6) on which MAIT is based, where the reconstruction noise is almost eliminated entirely except for a very small amount on the centre line. Using Legendre polynomials to restrict the shape of the derivative image, fMAIT reduces this noise even further to a single spot at the centre, as can be seen in the lower right-hand panel. The remainder of this publication will investigate the performance of MAIT and fMAIT on both simulated and real experimental data. First, the ability for MAIT and fMAIT to handle varying image quality (*i.e.* signal levels and background noise) is systematically tested. We then show how MAIT and fMAIT have a unique advantage in the way they handle (and effectively eliminate) any background noise present in the image data. Some typical reconstruction times for MAIT and fMAIT are also provided, as well as an assessment of the achievable radial resolution of our approach. As a final definitive test of MAIT, we analyse high-resolution O_2_ photodissociation images,^62^ and scattering images for collisions between velocity controlled NO radicals and Ne or Ar atoms;^55^ in each case drawing comparisons with other reconstruction approaches.

## III. Results

### A. Simulated images

**Fig. 4:**
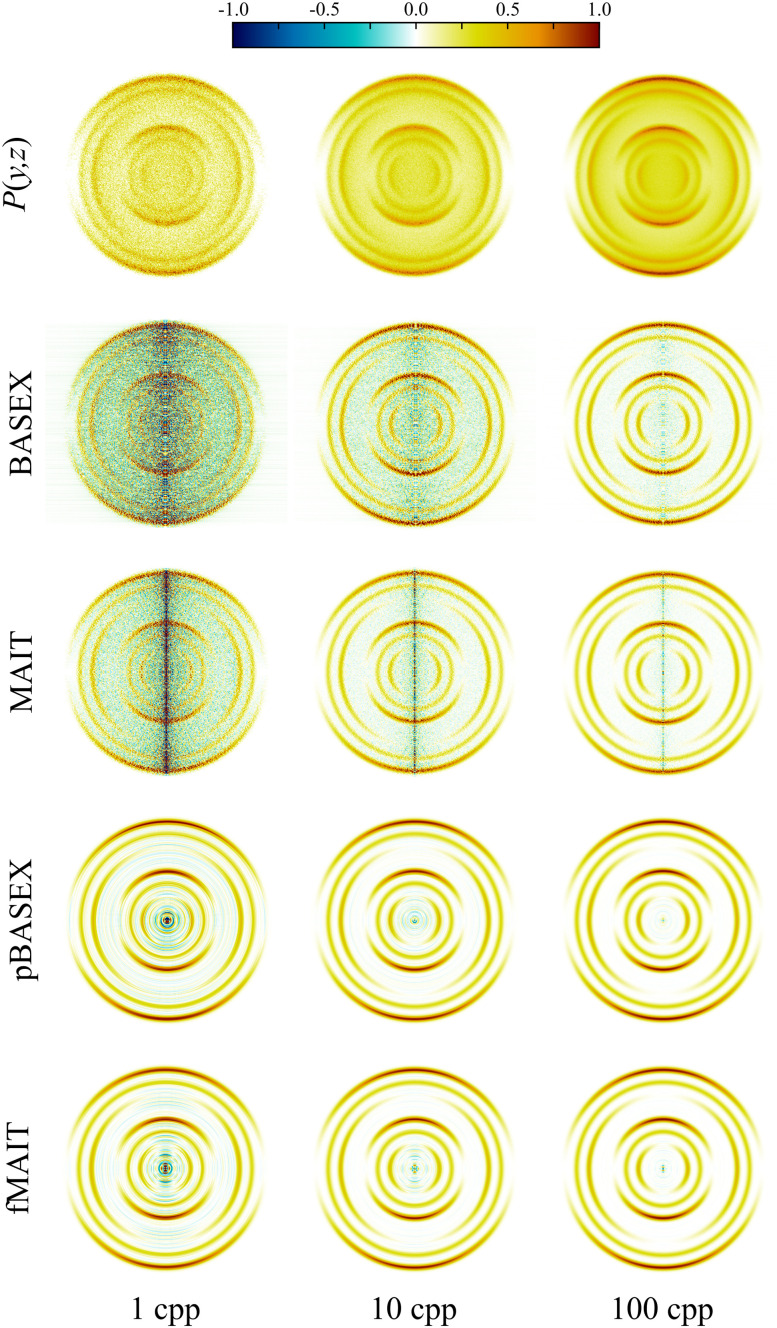
Simulated projection images with varying levels of signal noise (top row) and their corresponding reconstructions (the remaining four rows) using different Abel inversion techniques. No background noise contributions are included in these simulations. Visually, MAIT has similar performance to BASEX, but produces slightly sharper reconstructions with less overall background noise at lower signal levels. fMAIT performs near identically to pBASEX across all noise levels investigated. See main text for full discussion.

The performance of MAIT and fMAIT are evaluated by reconstructing distributions with varying levels of noise added. This is modelled by varying the average number of counts per pixel (cpp) sampled from a known perfect distribution. This distribution is comprised of a series of Gaussian rings each with a varying level of angular anisotropy. The anisotropy is modelled using the well-known equation:7
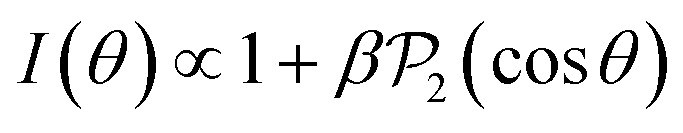
which is a special case of eqn (4). This form of angular distribution is widely applicable to studies involving ion-imaging of photodissociation and fragmentation,^2,6^ and also for photoelectron angular distributions resulting from a single-photon ionisation,^48^ making it an ideal first test for our proposed new reconstruction methods. Here, *θ* is the angle between the emitted photoproduct and the polarisation vector of the ionizing radiation, and *β* is the angular anisotropy parameter with limiting values of −1 and 2 (for perpendicular and parallel electric dipole transitions, respectively). Random statistical samples were drawn using the Poissonian imnoise filter in MATLAB onto a 512 × 512 pixel grid. In this section, image signal levels of 1, 10 and 100 cpp are considered. For sparser VMI data (with signal levels around 0.1 cpp or lower), the treatment of the projection image as a distribution (as is required to calculate its derivative) may begin to break down. In these cases, more advanced reconstruction procedures based on maximum-entropy reconstruction are required that can directly take into account the Poissonian nature of the individual ion/electron strikes that the image is composed of,^34–36^ and MAIT/fMAIT (and to some extent, BASEX) will not formally be suitable. The simulated images are shown in the top row of Fig. 4. The symmetry axis of these images lies in the vertical direction, with *θ* = 0° at the top of the image. Beneath each of these simulated projection images, reconstructions are shown using (from top to bottom) BASEX, MAIT, pBASEX or fMAIT. It is then easy to compare and identify qualitative differences between each of the reconstruction approaches. Links to download the specific MATLAB implementations of BASEX and pBASEX used in this work may be found here.^50^

Starting with the ‘unfiltered’ BASEX and MAIT methods; BASEX has the option of adding an additional regularisation parameter to the reconstruction procedure. As discussed briefly earlier, this regularisation effectively damps the system of linear equations involved in the inversion and results in the reduction of noise toward the centre of the reconstruction image, with only a slight compromise in the final resolution. Compared with MAIT, BASEX does indeed present with less of a centre line artefact, but at the cost of some additional background noise being introduced, which is visible between the reconstructed rings in the VMI reconstruction data. Thus, although the reconstruction noise levels using MAIT may be slightly larger at the centre line when compared to BASEX, they are noticeably smaller throughout the remainder of the inverted image.

Next, comparisons can be drawn between the ‘filtered’ methods pBASEX and fMAIT, where some general form of angular distribution is assumed *a priori* during the reconstruction procedure. Here, the angular distribution in the reconstructions is constrained to follow eqn (7). As can be seen in the pBASEX and fMAIT rows of Fig. 4, this effectively removes the centre line artefact, and instead concentrates any reconstruction noise toward the very centre of the image. This extra angular fitting step helps produce smoother reconstructions, in particular on noisy data (see the 1 cpp column of Fig. 4). Each of the methods considered here produce qualitatively similar results. Although, considering the additional numerical complexities involved behind the scenes of the pBASEX reconstruction (*i.e.* the computation of a large basis set), it should be highlighted that fMAIT produces the same high-quality reconstructions at a minimal computational cost.

**Fig. 5:**
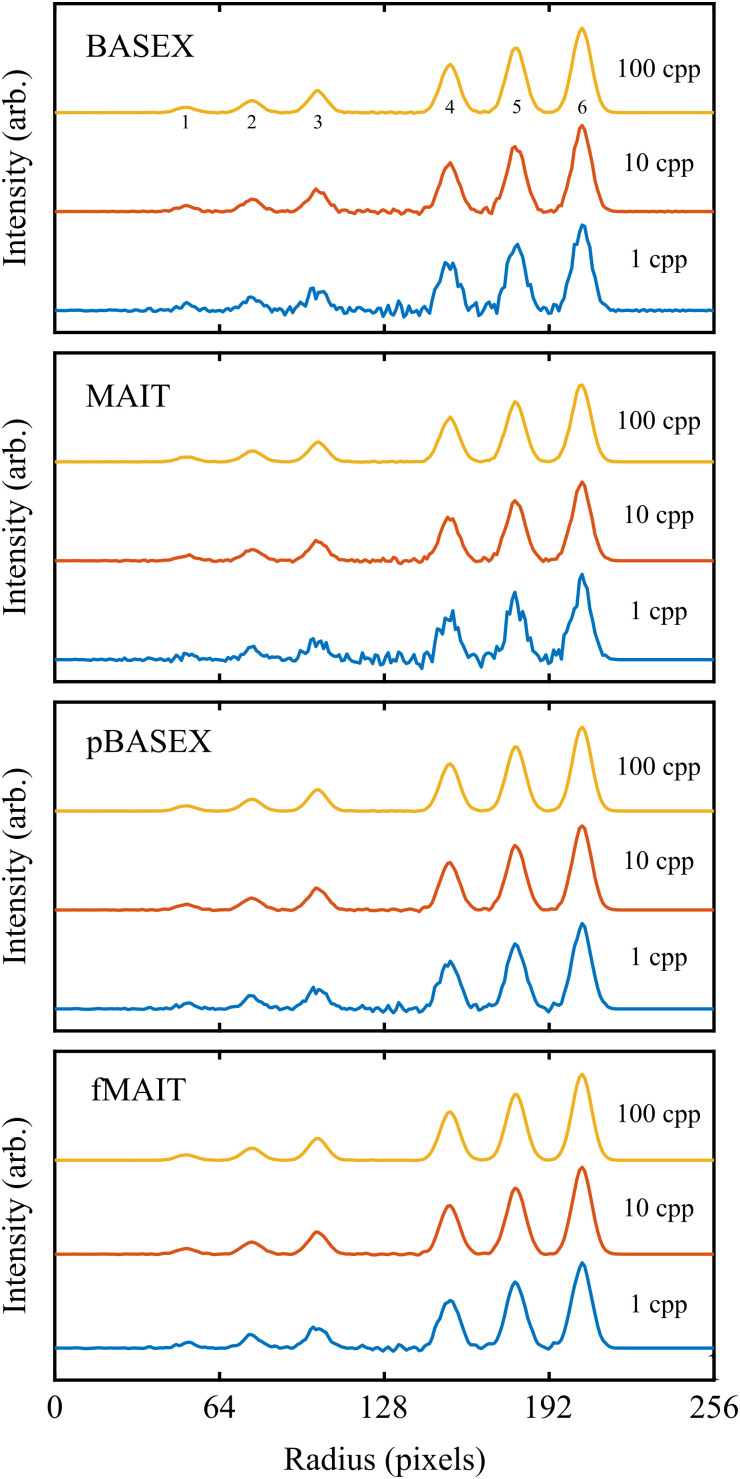
Angle-integrated velocity distributions for the different reconstructions displayed in Fig. 4, each of which is derived from a simulated VMI image with a signal level of either 1, 10 or 100 cpp. All reconstruction methods produce reasonable broadly similar results for low count rates, which improve as the count rate increases. The numbers in the top panel indicate the corresponding position of the rings in Fig. 3. See main text for full discussion.

More quantitative comparisons between the reconstruction approaches may be revealed by instead extracting the spectroscopic content available from these VMI images. The angle-integrated velocity distributions obtained from each of the reconstructed images (shown in Fig. 4) are plotted in Fig. 5. Perhaps the most notable thing about these plots is that the simulated signal level and subsequent reconstruction noise has very little effect on the retrieved velocity distribution. This is due to the *r*sin(*θ*) Jacobian weighting that must be applied to the reconstruction during the angle integration procedure. This is a general feature of the integral nature of calculating velocity distributions and highlights the fact that they are not highly sensitive to the reconstruction method used.

The retrieved angular anisotropy parameters, however, are a somewhat more sensitive test of the inversion procedure and are more susceptible to reconstruction noise. These are displayed in the upper half of Table 1. For each combination of signal level and reconstruction technique, six values are given corresponding to the mean anisotropy parameter value averaged over the full-width half-maximum (FHWM) of each peak in the velocity distribution. As the simulated signal levels increase (*i.e.* moving down the table), the retrieved *β* values generally approach the original simulated values (shown in the leftmost column) with the error bounds also decreasing for all reconstruction approaches. The BASEX and MAIT methods show similar performance to each other; as do the pBASEX and fMAIT methods. This reflects the same behaviour already observed in the reconstructed images (Fig. 4) and the velocity distributions (Fig. 5). Thus, we can conclude that for the reconstruction of typical images formed in photodissociation and photoelectron angular distributions studies, our two new methods MAIT and fMAIT perform at least as well as the most popular alternatives over a range of experimental data quality.

**Table 1:** Reconstructed values for the *β* angular anisotropy parameter for the images shown in Fig. 4 and 6. For each simulated signal or background noise level and each reconstruction method, the *β* value averaged over the full-width half-maximum (FWHM) of each peak in the corresponding velocity distribution is shown. The error bounds denote the 1σ standard deviation of the mean value across the FWHM. For the varying signal levels, all methods produce results in reasonable agreement with the initial values. For the background noise cases, however, MAIT and fMAIT show a much better agreement with the original simulated values compared to BASEX and pBASEX, where the anisotropy is consistently underestimated for high background noise levels

	Ring	Simulation	BASEX	MAIT	pBASEX	fMAIT
1 cpp signal	1	−1	−0.75 ± 0.20	−1.18 ± 0.20	−1.02 ± 0.11	−1.03 ± 0.09
	2	0	0.05 ± 0.14	-0.07 ± 0.21	−0.01 ± 0.07	0.08 ± 0.07
	3	2	1.99 ± 0.10	2.04 ± 0.17	2.03 ± 0.15	2.07 ± 0.04
	4	−1	−0.98 ± 0.08	−0.94 ± 0.07	−0.94 ± 0.07	−1.01 ± 0.04
	5	0	0.06 ± 0.04	−0.10 ± 0.08	0.03 ± 0.05	0.01 ± 0.03
	6	2	1.94 ± 0.07	1.91 ± 0.08	2.09 ± 0.10	2.02 ± 0.05

10 cpp signal	1	−1	−1.04 ± 0.06	−1.05 ± 0.05	−1.00 ± 0.03	−0.99 ± 0.04
	2	0	0.11 ± 0.05	0.05 ± 0.05	0.03 ± 0.02	0.04 ± 0.03
	3	2	1.97 ± 0.02	1.97 ± 0.03	1.98 ± 0.02	1.99 ± 0.02
	4	−1	−1.00 ± 0.02	−1.02 ± 0.03	−1.00 ± 0.02	−1.00 ± 0.02
	5	0	−0.01 ± 0.01	0.03 ± 0.02	−0.03 ± 0.01	0.00 ± 0.01
	6	2	1.99 ± 0.01	1.95 ± 0.02	2.00 ± 0.01	2.02 ± 0.01

100 cpp signal	1	−1	−0.98 ± 0.02	−0.95 ± 0.02	−0.98 ± 0.02	−0.97 ± 0.01
	2	0	−0.01 ± 0.02	0.04 ± 0.02	0.00 ± 0.01	0.03 ± 0.01
	3	2	1.97 ± 0.01	2.02 ± 0.01	2.01 ± 0.02	2.02 ± 0.01
	4	−1	−0.99 ± 0.01	−0.97 ± 0.01	−0.99 ± 0.01	−0.99 ± 0.01
	5	0	−0.01 ± 0.01	0.02 ± 0.01	−0.01 ± 0.01	0.00 ± 0.01
	6	2	1.98 ± 0.01	1.99 ± 0.01	2.01 ± 0.01	2.01 ± 0.01

1 cpp background	1	−1	−0.99 ± 0.04	−1.05 ± 0.11	−0.97 ± 0.03	−1.03 ± 0.04
	2	0	0.04 ± 0.03	0.16 ± 0.07	0.07 ± 0.01	0.08 ± 0.05
	3	2	1.93 ± 0.04	2.07 ± 0.02	1.97 ± 0.02	2.04 ± 0.02
	4	−1	−1.00 ± 0.02	−1.01 ± 0.02	−0.97 ± 0.01	−0.98 ± 0.02
	5	0	−0.01 ± 0.02	0.01 ± 0.03	0.03 ± 0.01	−0.06 ± 0.03
	6	2	1.94 ± 0.02	1.98 ± 0.02	1.97 ± 0.02	2.06 ± 0.05

10 cpp background	1	−1	−0.92 ± 0.03	−0.99 ± 0.07	−0.90 ± 0.05	−1.00 ± 0.09
	2	0	0.03 ± 0.05	0.16 ± 0.05	0.03 ± 0.02	0.03 ± 0.04
	3	2	1.81 ± 0.05	2.02 ± 0.07	1.90 ± 0.04	2.05 ± 0.01
	4	−1	−0.91 ± 0.02	−1.00 ± 0.05	−0.92 ± 0.01	−1.02 ± 0.02
	5	0	−0.07 ± 0.03	−0.04 ± 0.03	0.01 ± 0.01	0.06 ± 0.04
	6	2	1.74 ± 0.04	2.00 ± 0.05	1.77 ± 0.03	2.15 ± 0.03

100 cpp background	1	−1	−0.60 ± 0.05	−1.19 ± 0.16	−0.57 ± 0.04	−1.10 ± 0.15
	2	0	−0.01 ± 0.07	0.19 ± 0.12	0.01 ± 0.07	−0.06 ± 0.14
	3	2	1.02 ± 0.05	1.98 ± 0.06	1.06 ± 0.06	1.79 ± 0.06
	4	−1	−0.59 ± 0.02	−0.89 ± 0.05	−0.48 ± 0.02	−1.16 ± 0.03
	5	0	−0.13 ± 0.02	−0.09 ± 0.06	0.01 ± 0.01	−0.28 ± 0.06
	6	2	0.85 ± 0.02	1.95 ± 0.08	0.89 ± 0.01	2.16 ± 0.09

**Fig. 6:**
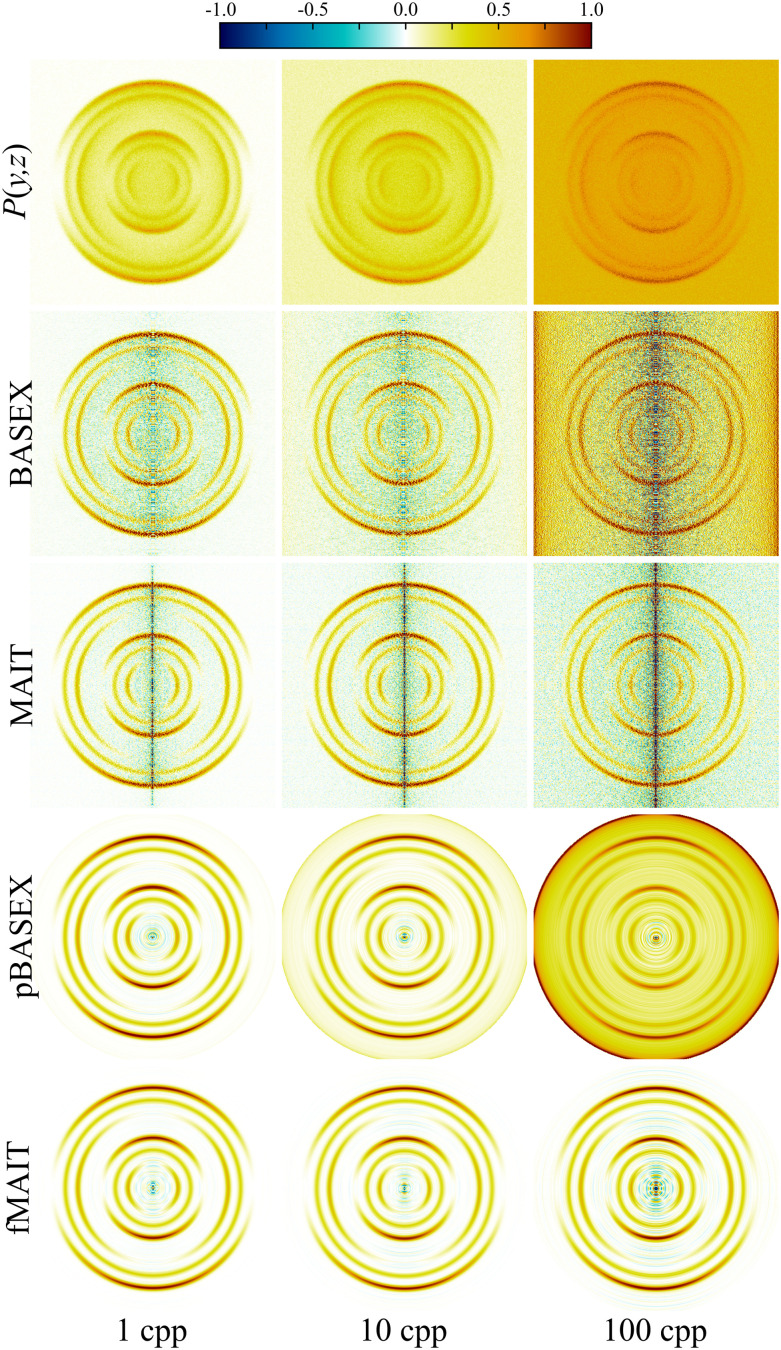
Simulated projection images with varying levels of background noise (top row) and corresponding reconstructions (the remaining four rows) using different Abel inversion techniques. The total signal level is held at 10 cpp in each case, with only the background noise contribution changing. For high background noise levels, both the BASEX and pBASEX reconstructions begin to display a great deal of background noise. This, however, is absent in the MAIT and fMAIT methods. See main text for full discussion.

One significant benefit to the new MAIT approaches introduced here, though, is how they handle *background* noise (*i.e.*, erroneous ion/electron strikes that are detected, but do not originate from the intended photoionization events). This can be effectively modelled as an additional random homogenous contribution across the image with an effective average cpp. For reasonably low levels of background noise, this noise will present as isolated Gaussian-like strikes on the otherwise empty background of the detector. In this regime, background noise has little effect on any extracted velocity distributions or angular anisotropy parameters. If increasing levels of background noise are present in images, however, most Abel inversion algorithms will attempt to also reconstruct the noise – a procedure with no physically sensible interpretation. In practice, efforts are usually made to subtract away low and constant levels of noise *via* some form of background subtraction. However, during the initial differentiation step of MAIT and fMAIT, rather pleasingly, any homogenous background noise vanishes since the derivative of this contribution is naturally zero. This benefit of our approach is highlighted in Fig. 6, by comparing reconstructions (once again produced using BASEX, MAIT, pBASEX and fMAIT) of simulated data with varying levels of background noise. In these examples, the signal level is held constant at 10 cpp (the case shown in the centre column of Fig. 4), while the background noise is now set to 1, 10 or 100 cpp on average. As can be clearly seen in Fig. 6, for increasing levels of background noise in the projection image data, a corresponding increase in positive background noise is seen in the BASEX and pBASEX reconstruction images. For MAIT, the background noise present in the reconstruction also increases with the cpp value of the projection image, but this noise is comparatively small and (as can be seen from the colourmap) fluctuates between positive and negative values. Therefore, it is not expected to contribute significantly when integrated to find the velocity distributions or when angular distributions are extracted, both of which will be discussed shortly. The fMAIT reconstructions present with the least amount of reconstruction noise. Any noise that is not filtered out during the derivative step is taken care of during the Legendre expansion, leading to very qualitatively appealing reconstructions across all simulated levels of background noise.

**Fig. 7:**
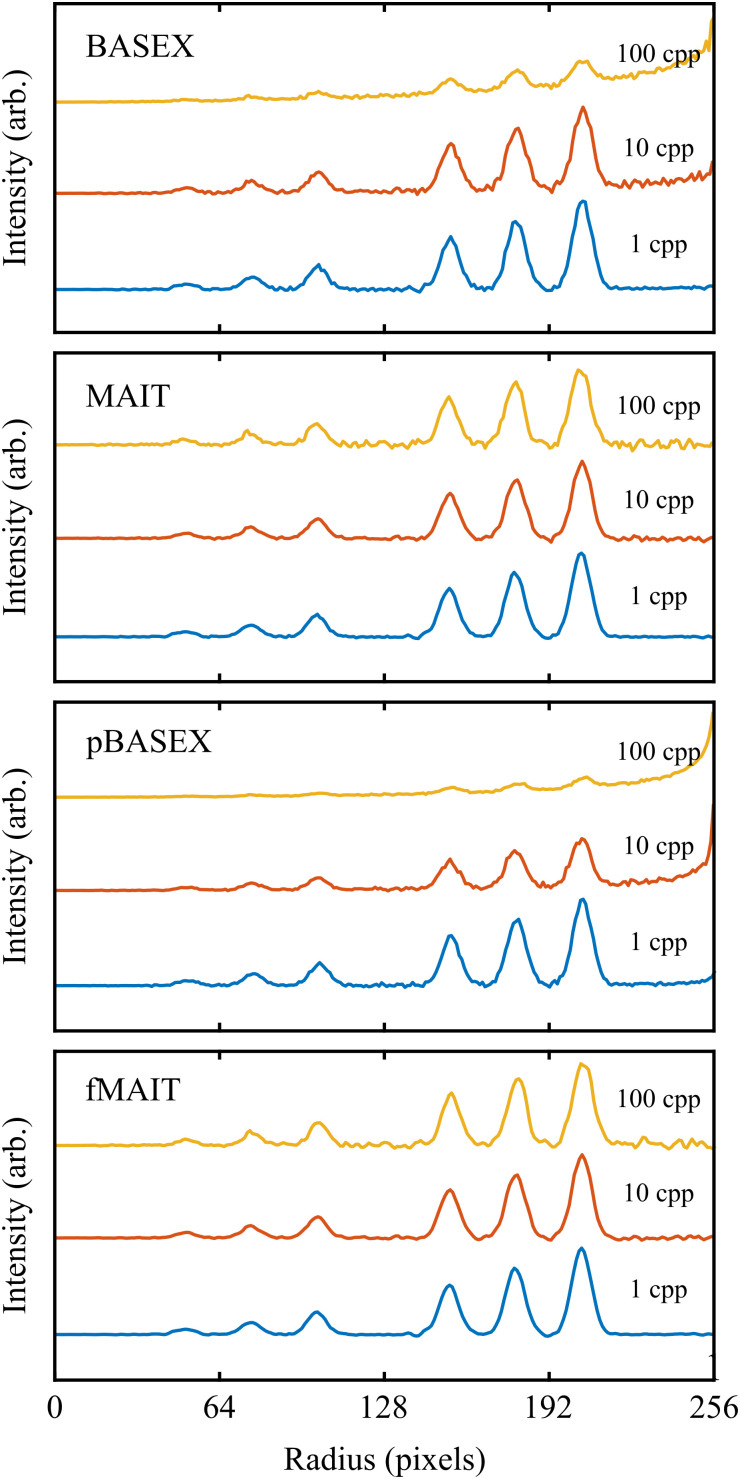
Angle-integrated velocity distributions for the different reconstructions displayed in Fig. 6, each of which is derived from a simulated VMI image with a background noise level of either 1, 10 or 100 cpp. BASEX and pBASEX perform the poorest here, with peaks being less well resolved and a background artefact appearing at large radii for larger background noise levels. The derivative step in our new method, however, filters away most of the background noise contribution and so it does not significantly effect the retrieval of velocity distributions.

Analogous to the previous noise test, angle-integrated velocity distributions and angular anisotropy parameters can be calculated from the reconstructions. These are shown in Fig. 7 and in the lower entries of Table 1, respectively. As was anticipated given the reconstruction images, both BASEX and pBASEX produce distributions with an erroneous background feature extending out to large velocities (radii). On the other hand, this background feature is completely absent in the MAIT and fMAIT reconstructions. Interestingly, this benefit of MAIT/fMAIT would also apply in cases where the apparent ‘background’ contribution actually originates from high velocity components, with a velocity corresponding to a radius larger than that of the imaging detector. In this case, the corresponding peak in the velocity distribution would not be accessible experimentally, but it would be known to fall outwith the velocity range captured by the detector and have no influence on the slower components that are detectable; exactly as is recovered using MAIT and fMAIT. For the case of the extracted angular distributions, the background noise also introduces further problems for BASEX and pBASEX. The background offset present in these images effectively reduces the anisotropy in the data, resulting in *β* values that are consistently *smaller* than those originally simulated for BASEX and pBASEX at higher background noise levels. In contrast, the MAIT and fMAIT approaches are again more robust to the simulated background noise, and in most cases, *β* values are extracted that are in remarkably good agreement with the original simulations, given the background noise level present.

### B. Timing comparisons

**Fig. 8:**
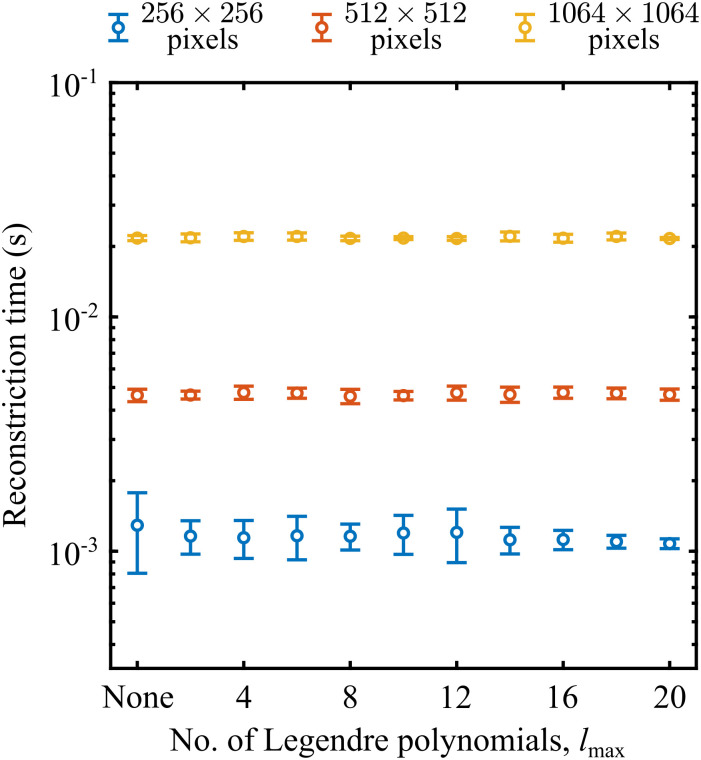
A comparison of reconstruction times using MAIT (for the ‘none’ case) and fMAIT (for the remaining points) for different image sizes and for different polynomial expansion sizes. Times are produced by performing 10 reconstructions for each image size and polynomial combination and averaging the result. Error bars denote the 1σ uncertainty in this mean value.

Reconstruction times can vary dramatically across different Abel inversion algorithms. More complex algorithms (particularly iterative algorithms that incorporate a more advanced statistical analysis of the data) are typically slower and invert images in a few seconds, whereas strategies that rely solely on linear algebra (as discussed in the introduction) are usually incredibly fast and are capable of processing megapixel images at rates of over one hundred frames per second.^31^ Typical reconstruction times for a variety of different image resolutions (shown as different coloured data points) and *l*_max_ values (plotted along the horizontal axis) are presented in Fig. 8 for MAIT/fMAIT. Timings were recorded on a 2023 Apple MacBook M2 Pro with 16 GB of RAM. Unsurprisingly, the reconstruction time is longer for larger images and scales proportionally with ∼*n*^2.5^ for an *n* × *n* pixel image – which is comparable to most other approaches.^31^ Even for relatively large images, however, the absolute reconstruction time is competitively fast and is perfectly suitable for real-time analysis applications, or for the analysis of large volumes of imaging data, such as is recorded in time-resolved photoelectron imaging experiments.^45,46^

As for the number of Legendre polynomials used in the fMAIT filtering step, we see perhaps more surprising behaviour. The rate-limiting step for our algorithm is in the calculation of the forward Abel transform of the intermediate image. In comparison to this step, the Legendre polynomial filtering step executes in an essentially negligible time. Therefore, there is really no difference in processing time when using the filtered fMAIT compared with the standard MAIT method. This is different from what is seen in other Abel inversion strategies which offer a ‘fitted’ and ‘non-fitted’ option, where typically the fitting step significantly extends the total reconstruction time.^40^ Furthermore, the threshold value of *l*_max_ also has no bearing on the total reconstruction time. This can be seen clearly in Fig. 8, since for each image size the reconstruction times for different *l*_max_ all lie on a horizontal line. When using pBASEX, to include higher order terms in the reconstructed angular distribution, a new basis set must be constructed from scratch and inverted each time (*i.e.* you cannot simply add terms to a pre-existing basis set). This can potentially be extremely time consuming (as has been highlighted elsewhere^51^) particularly for high-resolution images requiring correspondingly high-resolution basis sets. Our new approach, though, is far more user friendly – only requiring the change of a single number in the analysis with no knock-on effects for the reconstruction time. This makes this approach particularly appealing in circumstances where larger values of *l*_max_ may be expected, such as in Coulomb explosion imaging,^13,61^ above-threshold ionization processes,^58–60^ and molecular scattering measurements.^55–57^

### C. Radial resolution

The use of the derivative in the MAIT/fMAIT procedure, as was mentioned in the introduction, may have an effect on its maximal possible reconstruction resolution. This is because the calculation of the numerical derivative necessitates replacing the real measured data with some approximation that can be differentiated. This is equivalent to introducing a small level of smoothing to the data, which will potentially result in problems when trying to reconstruct particularly fine and sharp features in VMI data. For many applications where features are several pixels wide – such as the examples already discussed in Section III A – this will be a negligible effect. Even for the high-resolution images that will be discussed later in Section III D and E, we see no significant influence of this in the reconstructed data. In the interest of full transparency, though, we illustrate here the consequences of using this numerical derivative approximation, and the mitigating effects it may have on the achievable radial resolution in extreme cases.

**Fig. 9:**
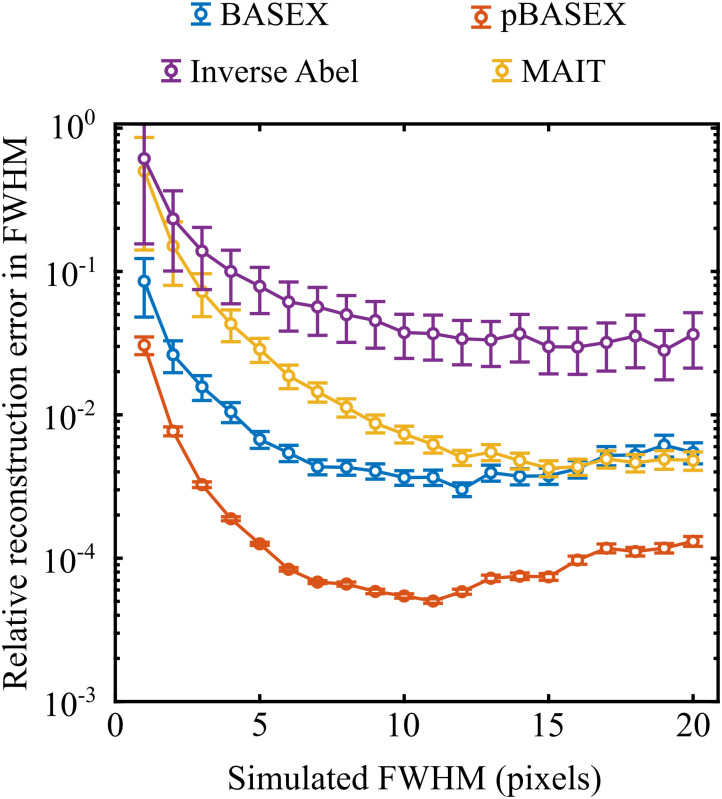
Relative errors in reconstructed FWHM values for different reconstruction approaches. The MAIT/fMAIT approach (yellow) consistently outperforms the simple implementation of the inverse Abel transform (purple). For widths greater than around 15 pixels, the derivative step in the MAIT/fMAIT procedure has a negligible additional effect on the reconstruction resolution, and the result is comparable to that of BASEX (blue).

Test VMI images were simulated consisting of three isotropic rings spaced 20 pixels apart with a signal level of 10 cpp (using the procedure established previously). The full-width half-maximum (FWHM) of each ring was varied as the input for this test from 1 to 20 pixels, and the corresponding integrated radial distributions were recovered using the inverse Abel integral, BASEX, pBASEX and MAIT. The reconstructed FWHM may then be easily compared with the original simulated FWHM value to assess the inherent radial resolution of each approach. We do this here by convoluting the simulated velocity distribution with a Gaussian of variable width and performing a least-squares minimization search to find the convolutional width which is in best agreement with the reconstructed data. This may then be used to find the actual reconstructed peak FWHM for each method, and the relative difference from the simulated input FWHM value may be determined. Fig. 9 shows a plot of how this error changes as the simulated FWHM is varied.

With this analysis, pBASEX gives the lowest reconstruction error for all simulated peak widths, followed by BASEX. The MAIT (and fMAIT – not shown as it performs identical to MAIT in this test) method consistently outperforms the standard inverse Abel transform; likely because of the significant reduction in the centre line artefact with MAIT. For particularly narrow features (*i.e.*, FWHM < 15 pixels) however, MAIT has a larger reconstruction error than that of BASEX. This is attributed to the small degree of smoothing that must be inherently present due to the differentiation step. For wider features, though, the MAIT and BASEX error curves converge and overlap each other, indicating that this smoothing becomes negligible for features wider than *ca.* 15 pixels. Similar trends were also noted for other signal levels, though the point at which the reconstruction models converge moved to larger simulated FWHM values for higher signal levels.

Though this may place a limit on the use of MAIT/fMAIT for particularly high-resolution imaging applications, for general use cases we anticipate the effects to be negligible (as can be seen in Section III A). We also would like to stress that the differentiation has no effect on the *angular* resolution of the reconstructions, as shall be demonstrated and discussed in more detail in Section III E.

### D. Photodissociation of O_2_

**Fig. 10:**
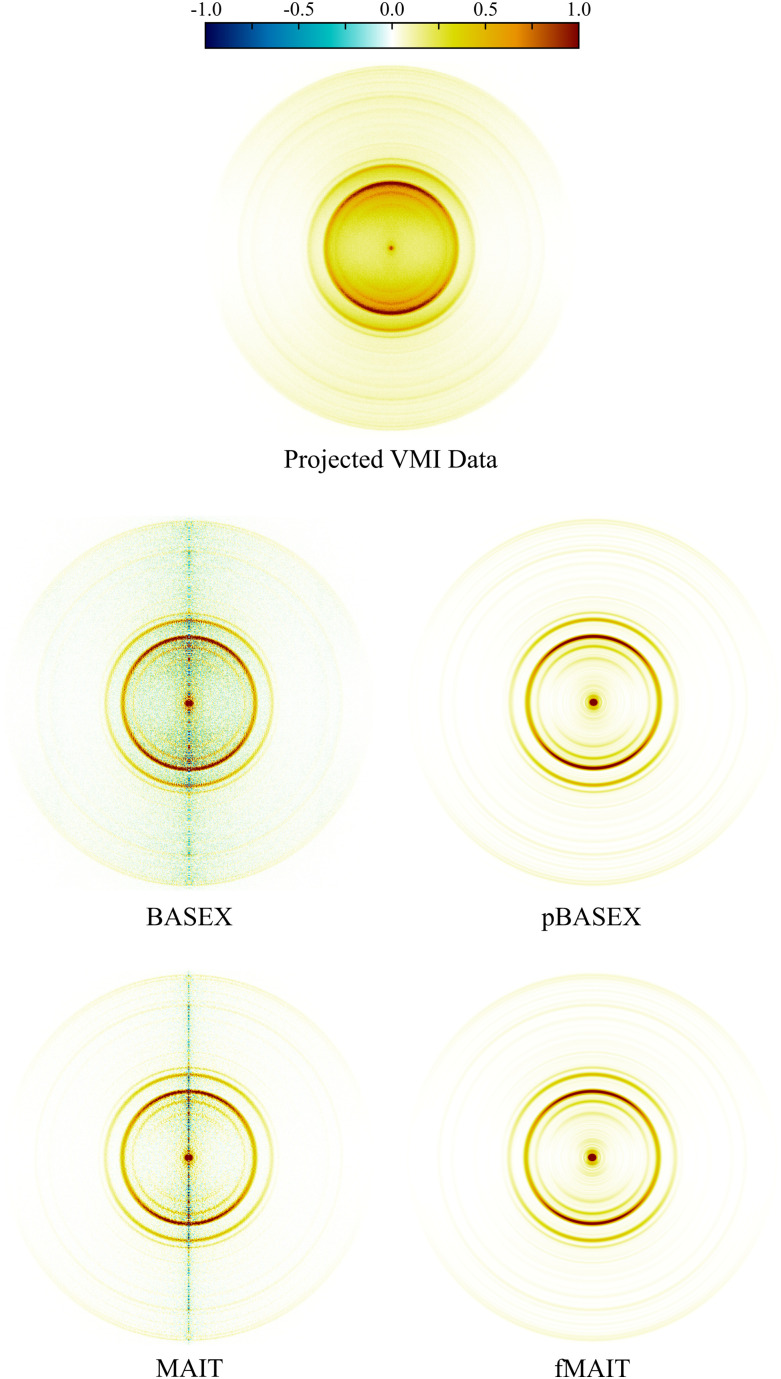
Raw (top panel) and reconstructed (rest) VMI data of O^+^ ions from the photodissociation of O_2_*via* the 3dπ 
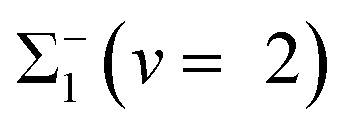
 Rydberg state using various reconstruction approaches. See main text for discussion. An extended discussion if this data may be found elsewhere.^62^

In these final two sections, the abilities of MAIT and fMAIT are showcased with demonstration reconstructions of experimental VMI images. For the first example, the image in the top panel of Fig. 10 comes from a recent publication focussing on the photodissociation dynamics of O_2_*via* two-photon resonant Rydberg states in the 200–240 nm region.^62^ O_2_ photodissociation was also used in the seminal demonstration of the VMI technique by Eppink and Parker in 1997,^16^ and so is a highly appropriate test system for the initial use of MAIT and fMAIT presented in this article. The particular image shown in Fig. 9 is obtained by imaging O^+^ ions formed by exciting O_2_*via* the 3dπ 
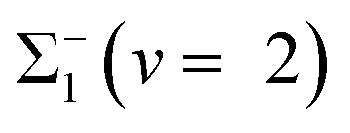
 Rydberg state at 224.983 nm – see ref. 62 for full details.

As was done in the previous section, the respective outputs of MAIT and fMAIT are also compared with BASEX and pBASEX. The reconstructions produced using BASEX and pBASEX are shown in the centre panels of Fig. 10, with the MAIT and fMAIT reconstructions shown in the lower panels. As was the case for the test imaging data presented in Section III A, the BASEX reconstruction has a slightly reduced central noise line compared to the MAIT reconstruction at the cost of a larger oscillating background contribution (note the green/yellow colour dispersed over the image). This background reconstruction contamination is almost entirely absent from the MAIT reconstruction, with the vast majority of any reconstruction artefacts lying at the symmetry axis. Also in analogy with the simulated examples discussed earlier, the pBASEX and fMAIT reconstructions are near identical as a consequence of forcing the inverted images to obey eqn (7).

**Fig. 11:**
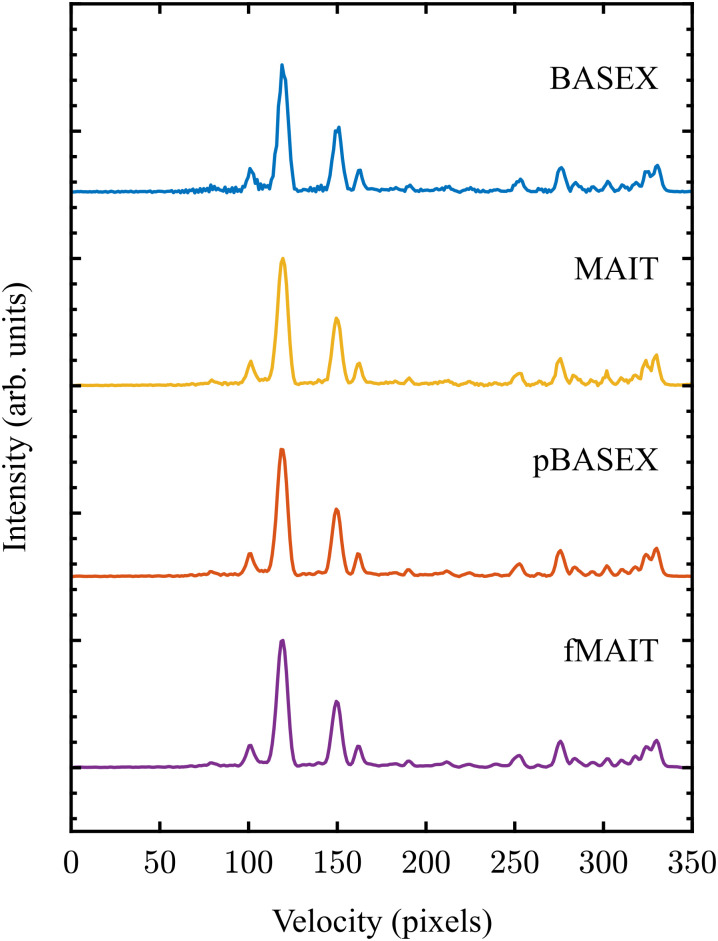
Velocity distributions extracted from the reconstructed images shown in Fig. 10. Due to the high quality of the raw experimental data (also Fig. 10), the retrieved velocity distributions are essentially identical.

Fig. 11 shows the velocity distributions obtained by angular integration of each reconstructed image in Fig. 10. Despite the small variation in image reconstruction quality seen in Fig. 10, there is no appreciable difference between the velocity distributions. This again demonstrates the robust nature of the angular integration process within any small levels of reconstruction noise. As stated earlier, there is no discernible difference between the output of fMAIT and pBASEX. Note again, though, that the fMAIT reconstruction does not require the pre-calculation of a 2D basis set, unlike pBASEX. Thus, fast, high-resolution, basis set-free reconstructions are made possible using the fMAIT approach.

### E. NO–Ne and NO–Ar scattering images

As a final and most challenging test of MAIT and fMAIT, we consider VMI images recorded from atom–diatom scattering measurements. These are an excellent proving ground for new data reconstruction strategies, since they can often contain rich features which are challenging to resolve.^55^ In general, however, crossed-beam scattering VMI data is not Abel invertible. This is due to kinematic effects between the two collision partners, resulting in a pixel-dependent so called flux-to-density transformation which effectively breaks the cylindrical symmetry prerequisite for an Abel inversion. As such, the inverse Abel transform is rarely used for interpreting scattering data and instead more advanced image reconstruction procedures must be used.^26–29^ For the specific cases of ‘head-on’ collisions with counterpropagating molecular beams or for perfectly merged beams, however, flux-to-density effects are uniform across the detection plane and cylindrical symmetry is preserved in the scattering images – allowing for the data to be reconstructed *via* Abel inversion. These particular collision geometries – in combination with the Stark deceleration technique – also minimize the effects of the velocity spread of each beam on the final angular resolution possible in the imaging data. This allows for the finest of details present in scattering differential cross-sections (DCSs), such as diffraction oscillations,^55^ to be measured with exquisite precision. Diffraction oscillations are a result of quantum interference between different collision trajectories across the interaction potential energy surface which result in the same final deflection angle.

**Fig. 12:**
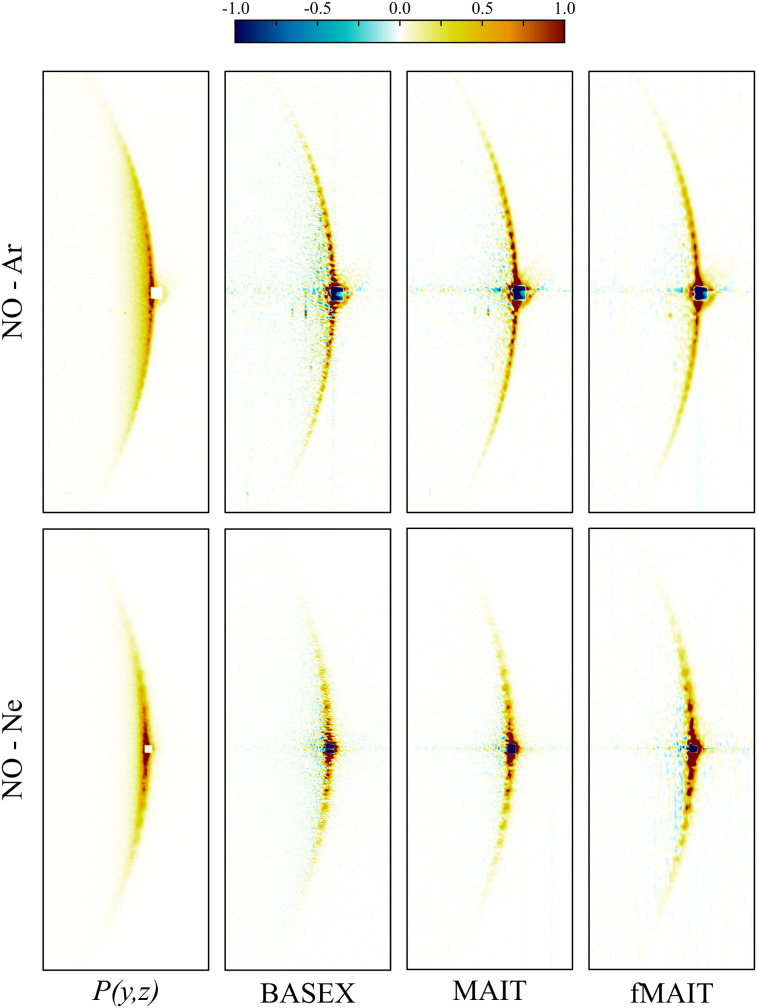
Cropped views of NO ion images recorded following inelastic scattering with Ar (top row) and Ne (bottom row). The original VMI data is shown on the far left and the various reconstructions are shown on the right. In these images, the NO velocity vector points from left to right. Small areas of the distributions around the forward scattering direction are masked due to imperfect state selection of the NO packet. While all inversion approaches clearly resolve the diffraction oscillations present in the original data, there is slightly less background reconstruction noise dispersed over the MAIT/fMAIT images. See main text for full discussion. A detailed discussion and extended analysis of this data is available elsewhere.^55^

Segments of ‘head-on’ collision images recorded in a previous study of the inelastic scattering of NO with rare gas atoms (Ar and Ne) are shown in Fig. 12.^55^ For both cases, the image has been reconstructed using BASEX (as was done in the original publication of this data) and using the new MAIT and fMAIT methods proposed in this article. pBASEX is not included here in this comparison since, as discussed earlier, constructing a 2D basis set capable of modelling the oscillations seen in this form of measurement would be prohibitively time consuming. If the extensive computer memory required was available, we tentatively estimate it would take in excess of 30 hours to construct a basis set with *l*_max_ = 720 (which allows for oscillations at the scale of ∼0.5° to be reconstructed). Furthermore, the system of linear equations to be solved with such a large basis set would be unstable and impractical to solve. The success of pBASEX (and also fMAIT) relies on being able to efficiently compress the VMI data with a simplified description in terms of a relatively small Legendre polynomial expansion. When this is no longer appropriate – as is the case for the images in Fig. 12 – there is no real advantage to these approaches, and in fact, the need to limit the angular resolution of the reconstructions with some *l*_max_ threshold can only spoil the otherwise achievable angular resolution. The fMAIT reconstruction (with *l*_max_ = 720) is therefore only shown here for completeness in Fig. 12 and to show that even angularly complex reconstructions are possible using fMAIT on reasonable time scales. It will not be considered further here.

Comparing the BASEX and MAIT approaches (the centre four panels of Fig. 12), it can be seen that there is a more diffuse background reconstruction noise present in the BASEX reconstructions (the yellow/green points just inside the rings). For the MAIT reconstructions, however, this noise is reduced and is instead only found much closer to the centre line where it is much less detrimental to the analysis of angular distributions. Integrating over each of the rings in Fig. 12, DCSs displaying the diffraction oscillations can be extracted as a function of *θ*, defined as the angle between the relative velocity vector of the two collision partners and the final velocity of the scattered products. In the images in Fig. 12, the right-hand side of the images corresponds to forward scattering (*θ* = 0°).

**Fig. 13:**
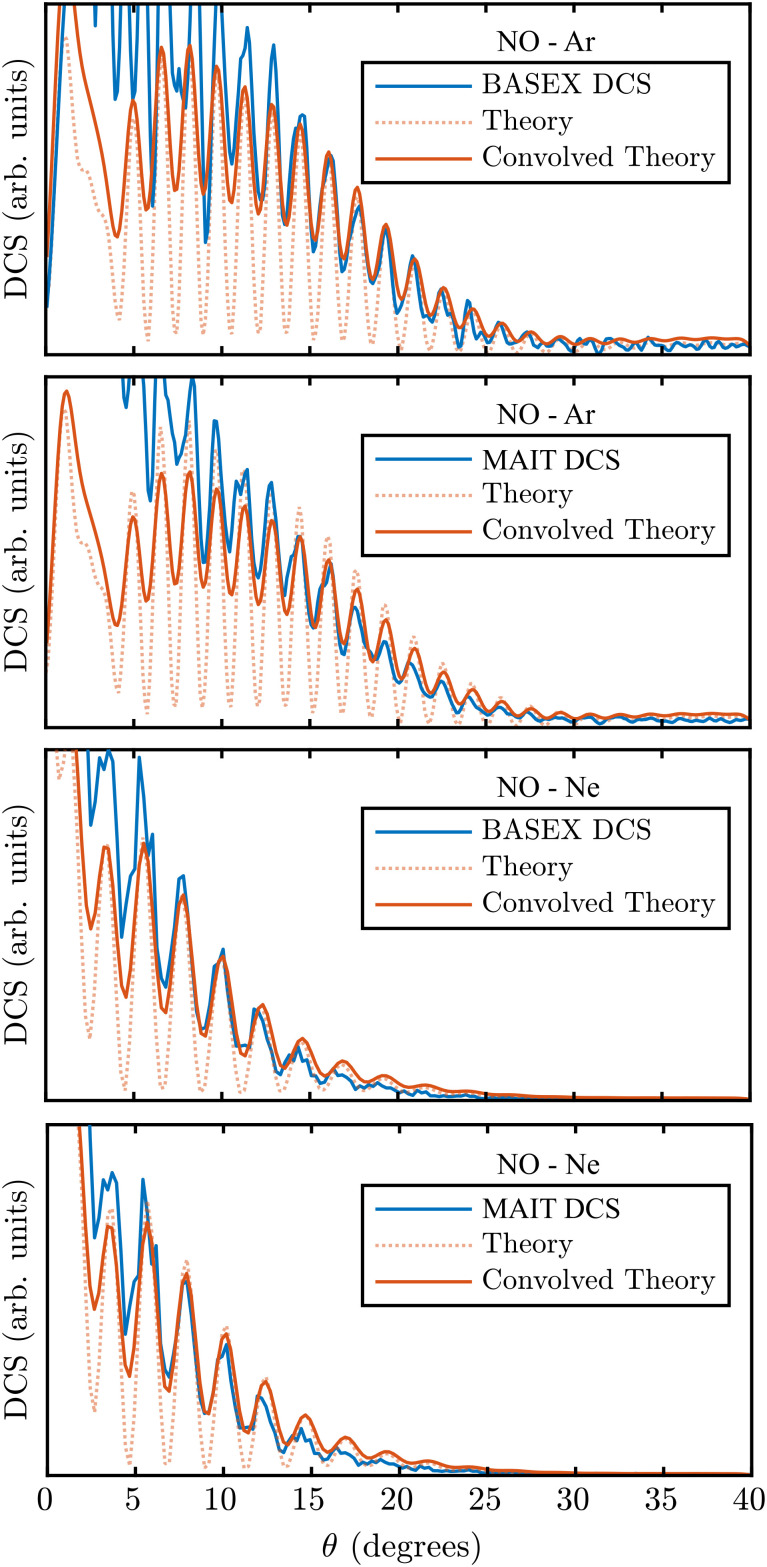
DCSs extracted from the scattering images shown in Fig. 12. For each combination of rare gas collision partner and reconstruction approach, the angular resolution has been extracted using a non-linear least-squares fitting procedure. For BASEX, angular resolutions of 0.35° and 0.42° were determined for Ar and Ne colliders, respectively. The optimal resolution possible using our new approach was found to be similar with 0.36° and 0.41°, respectively. This indicates that each reconstruction approach is capable of the same high- resolution, which is only limited by the imaging spectrometer itself.

Fig. 13 shows these extracted DCSs for NO–Ar and NO–Ne collisions extracted from the BASEX and MAIT reconstructions in Fig. 12 (blue lines). As with the images, clear oscillations can be observed using both reconstruction approaches. The full interpretation of these results can be found in the original publication.^55^ Here, instead, we focus on the relative resolution achieved with each reconstruction method. Also shown in Fig. 13 is the theoretical prediction for the DCS (red dotted lines). These oscillations are free from any resolution-limiting factors introduced either in the experimental imaging apparatus or from the reconstruction procedure. If the final resolution in the experimental DCSs reconstructed using either BASEX or MAIT are different, this will be due only to the inversion method. By convolving the theoretical DCSs with a Gaussian function of a variable width, the inherent resolution of the instrument combined with each reconstruction approach can be estimated by finding the Gaussian convolution width that best reproduces the experimental DCS – as was done previously in Section III C to investigate the radial resolution of each approach. These are shown overlaid in Fig. 13 as red solid lines. This fitting procedure retrieved an angular resolution of 1*σ* = 0.35° and 0.42° for the BASEX-reconstructed Ar and Ne collision images, respectively. For the MAIT images, a near-identical resolution of 1*σ* = 0.36° and 0.41° was retrieved from the same optimisation algorithm. This clearly demonstrates that our new method is a robust and valid alternative to BASEX, with the same high-resolution capabilities, and does not introduce any further detrimental effects on the final images and ultimate experimental result quality.

Confirming these high angular resolution image reconstruction capabilities of MAIT and fMAIT helps build confidence in these newly developed approaches. This, coupled with the competitively fast reconstruction times – especially for pBASEX-style reconstructions utilising large basis sets – and the unique ability to filter away background noise makes MAIT/fMAIT an excellent choice of Abel inversion approach across a broad range of VMI applications.

## IV. Conclusion

A new approach for reconstructing VMI projection data has been presented. This method uses a modified version of the inverse Abel transform integral, which has been mostly avoided due to its historical poor performance on experimental data. It has been shown here that by reframing the problem in terms of the more stable forward Abel transform, the majority of the reconstruction artefacts directed toward the centre of the image can be made to vanish. This is particularly useful for extracting photoproduct angular distributions and differential cross-sections. Some fine radial resolution is lost using the MAIT/fMAIT procedure when compared to other reconstruction approaches, though this is negligible in many cases and only becomes important for features that are only a few pixels wide. We have demonstrated here how our methods typically perform favourably against BASEX and pBASEX over a range of data quality and can even outperform these methods when a high level of unwanted background signal is present in the images – a feature we believe to be unique to the approach described here. This, in addition to the fast processing time, makes MAIT and fMAIT particularly attractive for on-the-fly reconstructions where signal-to-noise ratios may be poor. Our methods, however, may struggle to discern particularly sharp features in product velocity distributions when compared to alternative strategies. Although it is not discussed here, with some further modifications to the integral step, we also anticipate that this method will be readily applicable for the reconstruction and correction of partially sliced data.^40,41^ The MAIT/fMAIT MATLAB algorithm is available from the MATLAB File Exchange.^63^

## Conflicts of interest

There are no conflicts to declare.

## Supplementary Material

CP-027-D5CP00857C-s001

CP-027-D5CP00857C-s002

CP-027-D5CP00857C-s003

## Figures and Tables

**Fig. 14:**
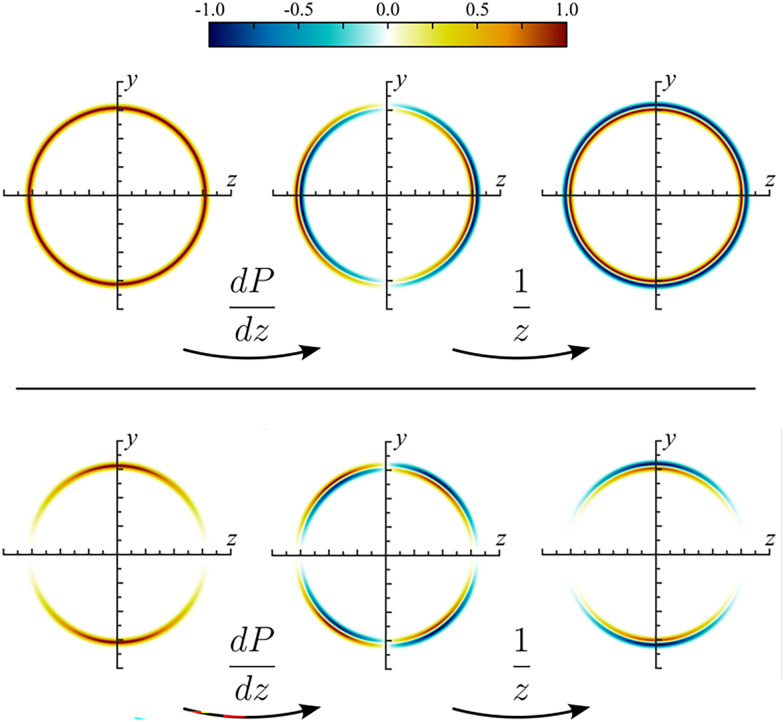
The first two steps of the MAIT reconstruction procedure applied to a model isotropic (top row) and anisotropic (bottom row) distribution. No singularities arise in the equations that produce these images, and this behaviour generalises to more complex shaped angular distributions.

**Fig. 15:**
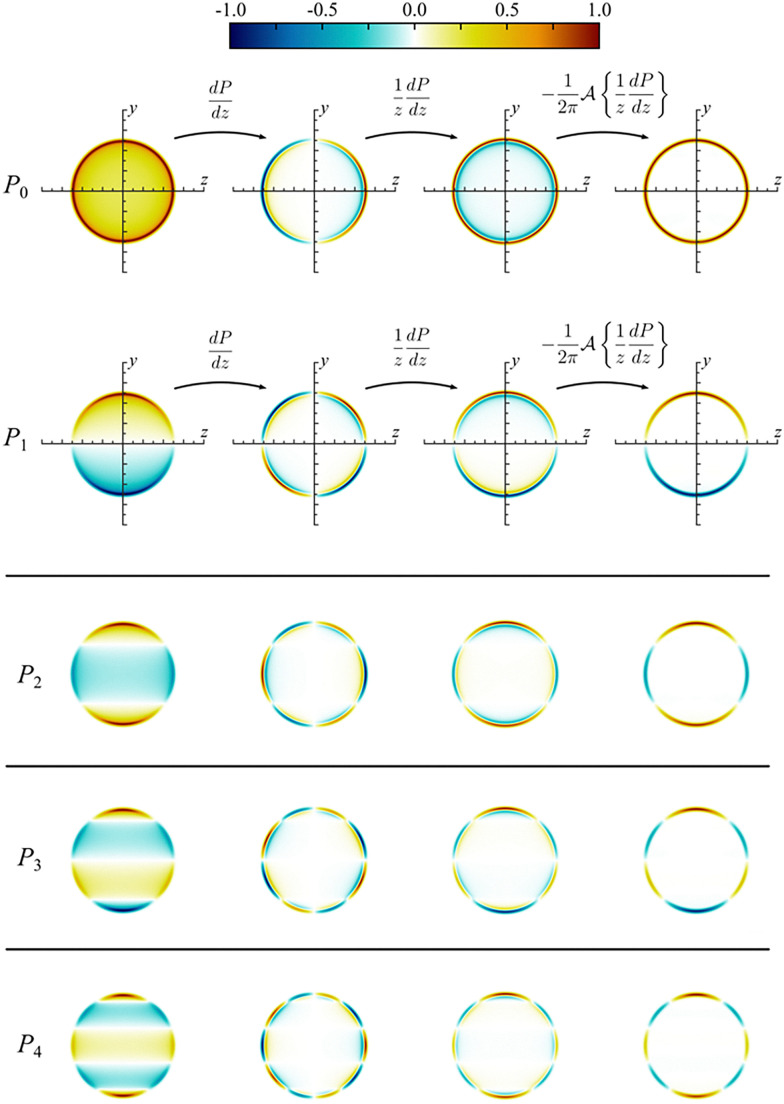
The MAIT procedure applied to projected angular distributions of different Legendre polynomials for *l* = 0 through to *l* = 4. For distributions with even symmetry (*i.e.* even values of *l*) the image produced in the derivative step (second column) can be expanded using only odd Legendre polynomials as a noise filtering step when using fMAIT. For odd symmetries (*i.e.*, odd *l*), the derivative image must instead be expanded in associated Legendre polynomials. The remainder of the reconstruction procedure is identical for both symmetries.

## Data Availability

The simulated data presented in Section III A along with the codes used to generate it are available from the authors upon reasonable request. For the experimental data examples used in Section III C and D were not generated for this work, and we direct interested parties to the original publications that present these data.^55,62^

## Appendices

### Appendix: additional mathematical details

Reframing the ill-conditioned inverse Abel integral as the forward Abel transform of a related function will only prove to be a robust approach if this new related function is well-behaved and does not contain any unphysical values (introduced by trying to divide by zero, for example). The goal of this Appendix is to verify that MAIT is on firm mathematical foundations, and does not produce any surprising artefacts. This can be demonstrated by first considering an isotropic Gaussian ring defined in the imaging plane, *P*(*y*,*z*):

A1

which is an example of the simplest structure present in VMI data, with width *w*_0_ and radial size *r*_0_. More complex VMI images can be constructed as a sum of this simple building block with different ring sizes and different degrees of angular anisotropy (to be discussed shortly). To differentiate eqn (A1) with respect to *z* (as is required in the MAIT procedure):

A2

the chain rule can be applied:

A3

Using the chain rule a second time:

A4

and simplifying:

A5

leads to:

A6

The important point from this result is that, once differentiated, the original isotropic distribution (*i.e.* only dependant on the radial distance ) now contains a term in *z*. This demonstrates why dividing the derivative image by *z* does not formally introduce any divide by zero errors, since *z* is already a factor within the derivative image. The *z* factor also coincides with the first order Legendre polynomial term, which explains the increase in the number of polynomials required to expand the derivative image compared to the original distribution by one. Although it should be possible to handle it formally, in practice, there are no instances of dividing by zero in the programming of MAIT or fMAIT. The image coordinate system is defined so that the extreme right/left of the image is defined as *z* = ±1, respectively. When processing an image with an even number of pixels (as is required by the matrix transform of Livingstone *et al.*), this will result in there being no pixel with coordinate *z* = 0, and therefore no divide by zero instances.

Fig. 14 shows examples of the derivative and division steps for this isotropic ring (top row) and also for an anisotropic ring with *β*_2_ = 2 (top row). The angular description of this differentiation step is more complicated than the original definition of the angular distribution [*i.e.* in eqn (4) of the main text], since the differentiation is made with respect to *z* but the angular annistropy is described in *θ*. Nevertheless, because of the requirement of cylindrical symmetry, the *y*-axis (*i.e.*, *z* = 0) must be a local extremum of the image, and the derivative will be equal to zero.

For images of *even* symmetry (*i.e.*, containing only even Legendre polynomial contributions), their derivative image will be of entirely odd symmetry and contain only odd Legendre polynomials, as discussed in the main text. Note now that the symmetry axis for this new expansion is the *z*- rather than the *y*-axis. For images containing odd terms, however (such as those encountered in scattering images and in photoelectron circular dichroism measurements), the description becomes more complicated. This is because there is no longer a symmetry between the upper and lower halves of the *yz*-plane. Instead, associated Legendre polynomials,  need to be used for the expansion, where *l* is set to *l*_max_ and *m* runs from 0 in integer steps through to *l*_max_. This is illustrated for projections of the first five Legendre polynomials in Fig. 15. In practice, the choice of using either the standard or associated Legendre polynomials enforces forward/backward symmetry or allows for forward/backward asymmetry, respectively, in the reconstructed images. With the polynomial expansion complete, the division by *z* will still result in a 0/0 indeterminate form along the symmetry axis, but this will always produce a finite answer since the derivative image already contains a factor of *z*, as has already been discussed.
