# Supplementary figures and images for: Revisiting the inverse Abel integral for reconstructing velocity-map images
Source: Phys Chem Chem Phys. 2025 Aug 20;27(35):18694–709. doi: 10.1039/d5cp00857c (PMC12366321; doi:10.1039/d5cp00857c)

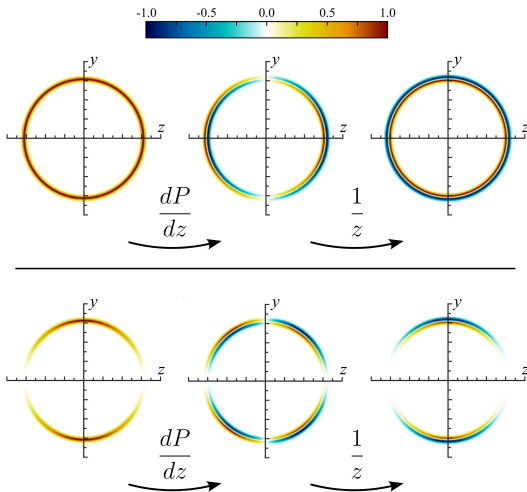

Supplement: CP-027-D5CP00857C-s001 [file CP-027-D5CP00857C-s001.pdf]

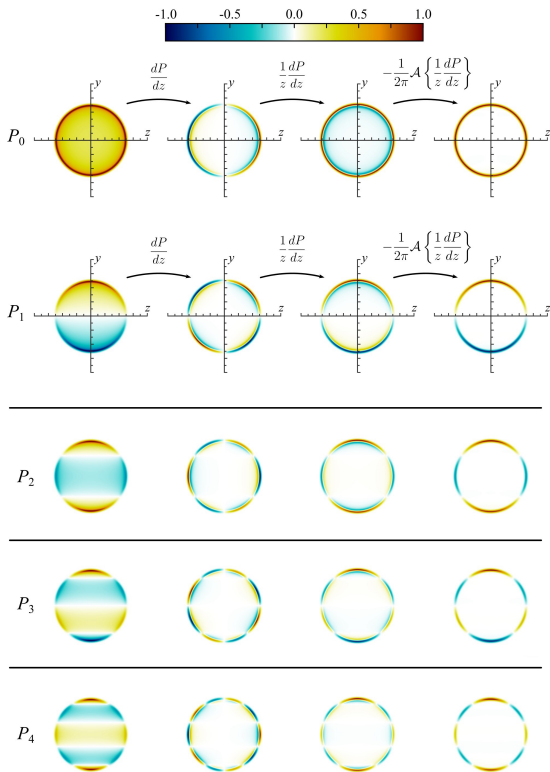

Supplement: CP-027-D5CP00857C-s002 [file CP-027-D5CP00857C-s002.pdf]
